# Melatonin-induced DNA demethylation of metal transporters and antioxidant genes alleviates lead stress in radish plants

**DOI:** 10.1038/s41438-021-00561-8

**Published:** 2021-06-01

**Authors:** Mingjia Tang, Liang Xu, Yan Wang, Junhui Dong, Xiaoli Zhang, Kai Wang, Jiali Ying, Cui Li, Liwang Liu

**Affiliations:** National Key Laboratory of Crop Genetics and Germplasm Enhancement, Key Laboratory of Horticultural Crop Biology and Genetic Improvement (East China) of MOAR, College of Horticulture, Nanjing Agricultural University, Nanjing, 210095 P.R. China

**Keywords:** Abiotic, Plant physiology

## Abstract

Melatonin (MT) is a tryptophan-derived natural product that plays a vital role in plant response to abiotic stresses, including heavy metals (HMs). However, it remains elusive how exogenous MT mediates lead (Pb) accumulation and detoxification at the methylation and transcriptional levels in radish. In this study, decreased Pb accumulation and increased antioxidant enzyme activity were detected under MT treatment in radish. Single-base resolution maps of DNA methylation under Pb stress (Pb200) and Pb plus MT treatment (Pb_50MT) were first generated. The genome-wide methylation level was increased under Pb stress, while an overall loss of DNA methylation was observed under MT treatment. The differentially methylated region (DMR)-associated genes between Pb_50MT and Pb200 were uniquely enriched in ion binding terms, including cation binding, iron ion binding, and transition metal ion binding. Hyper-DMRs between Pb200 and Control exhibited a decreasing trend of methylation under Pb_50MT treatment. A few critical upregulated antioxidant genes (e.g., *RsAPX2*, *RsPOD52* and *RsGST*) exhibited decreased methylation levels under MT treatment, which enabled the radish plants to scavenge lead-induced reactive oxygen species (ROS) and decrease oxidative stress. Notably, several MT-induced HM transporter genes with low methylation (e.g., *RsABCF5*, *RsYSL7* and *RsHMT*) and transcription factors (e.g., *RsWRKY41* and *RsMYB2*) were involved in reducing Pb accumulation in radish roots. These findings could facilitate comprehensive elucidation of the molecular mechanism underlying MT-mediated Pb accumulation and detoxification in radish and other root vegetable crops.

## Introduction

Contamination of soil and water with heavy metals (HMs) has become an increasingly concerning problem worldwide and affects human health through the food chain^1,2^. Lead (Pb), a widespread HM pollutant, is considered as a carcinogen and does not have any beneficial biological functions among all organisms. Lipid peroxidation, excessive reactive oxygen species (ROS), and DNA damage can be induced by Pb stress, resulting in the inhibition of plant growth and development^3^. Due to the transfer of Pb^2+^ from contaminated agricultural soil and irrigation water to food plants, several vegetable crops take up excess Pb^2+^ via different pathways, which potentially disrupts the nutrient balance in plants^4,5^. Hence, the reduction of Pb accumulation is imperative to effectively prevent toxicity in foods from crop plants.

DNA methylation, as an epigenetic modification, is associated with transcriptional activity and gene expression^6,7^. In plant genomes, methylated cytosines are categorized into three contexts: CG, CHG, and CHH (where H = A, T, or C). In *Arabidopsis*, CG and CHG methylation are maintained by a conserved DNA METHYLTRANSFERASE 1 (MET1) protein and CHROMOMETHYLASE3 (CMT3), respectively. Moreover, CMT2 and domain rearranged methyltransferase 2 (DRM2) primarily maintain CHH methylation^8,9^. In plants, DNA methylation levels are regulated not only by DNA methylation but also by demethylation reactions. In *Arabidopsis*, several DNA glycosylase/lyase enzymes critical for active DNA demethylation were identified, such as DEMETER (DME), REPRESSOR OF SILENCING 1 (ROS1), DEMETER-LIKE 2 (DML2) and DML3^10,11^. Recent studies have shown that the DNA methylation level is altered under a range of abiotic stresses, including HM and drought stresses^12,13^. In rice, most DNA methylation-modified genes are transcriptionally changed under cadmium (Cd) stress, suggesting that complex DNA methylation patterns have a direct relationship with stress responses and ultimately influence gene expression^12^. However, little information on genome-wide HM-induced DNA methylation patterns and features correlated with gene expression is available for radish.

Recent studies demonstrated that exogenous salicylic acid (SA), ethylene (ET), and melatonin could alleviate the adverse effects of HMs by reducing ROS and promoting HM detoxification^14–16^. Melatonin (N-acetyl-5-methoxytryptamine, MT) has emerged as a widespread and pleiotropic organic compound in various plants, such as *Arabidopsis*, cabbage, and cucumber^17–19^. MT plays vital roles in the response to HM stresses at the physiological and biochemical levels in plants^15,20^. For example, exogenous MT led to an increased tolerance to salt in rice and relieved Cd-induced damage in algae^21,22^. Nevertheless, the alteration patterns of Pb-induced DNA methylation and gene expression under MT treatment remain to be investigated in root vegetable crops, especially in radish.

Radish (*Raphanus sativus* L., 2*n* = 2*x* = 18) is an important annual or biennial root vegetable crop of the Brassicaceae family. Plant roots are considered as the most vulnerable tissue, with a direct correlation with the uptake of HMs from soil solution. It was reported that radish roots and hypocotyls accumulated a large amount of Pb, especially in roots, which accounted for almost 50% of the total lead content of the plant^23,24^. Hence, it is of great importance to reduce the Pb content and dissect the molecular mechanism of Pb detoxification in radish. Although several Pb-induced differentially expressed genes and microRNAs have been identified^25,26^, no systematic studies regarding MT-mediated DNA methylation changes under Pb stress have been conducted in radish. In this study, MT-induced dynamic changes in Pb content, plant growth, and antioxidant enzyme activities were measured. Whole-genome bisulfite sequencing (WGBS) was employed to generate genome-wide cytosine methylation maps with high coverage in radish. The purposes of this study were to explore the methylation change patterns and the relationship between the methylome and gene expression changes after Pb stress and MT treatment in radish. Furthermore, MT-induced differentially expressed genes (DEGs) and differentially methylated region (DMR)-associated genes under Pb stress were identified, and an MT-mediated regulatory network of Pb accumulation and detoxification was proposed. These results provide fundamental insights into the molecular mechanisms underlying MT-mediated Pb accumulation and detoxification in root vegetable crops.

## Results

### MT-induced reduction in Pb content in radish

**Fig. 1:**
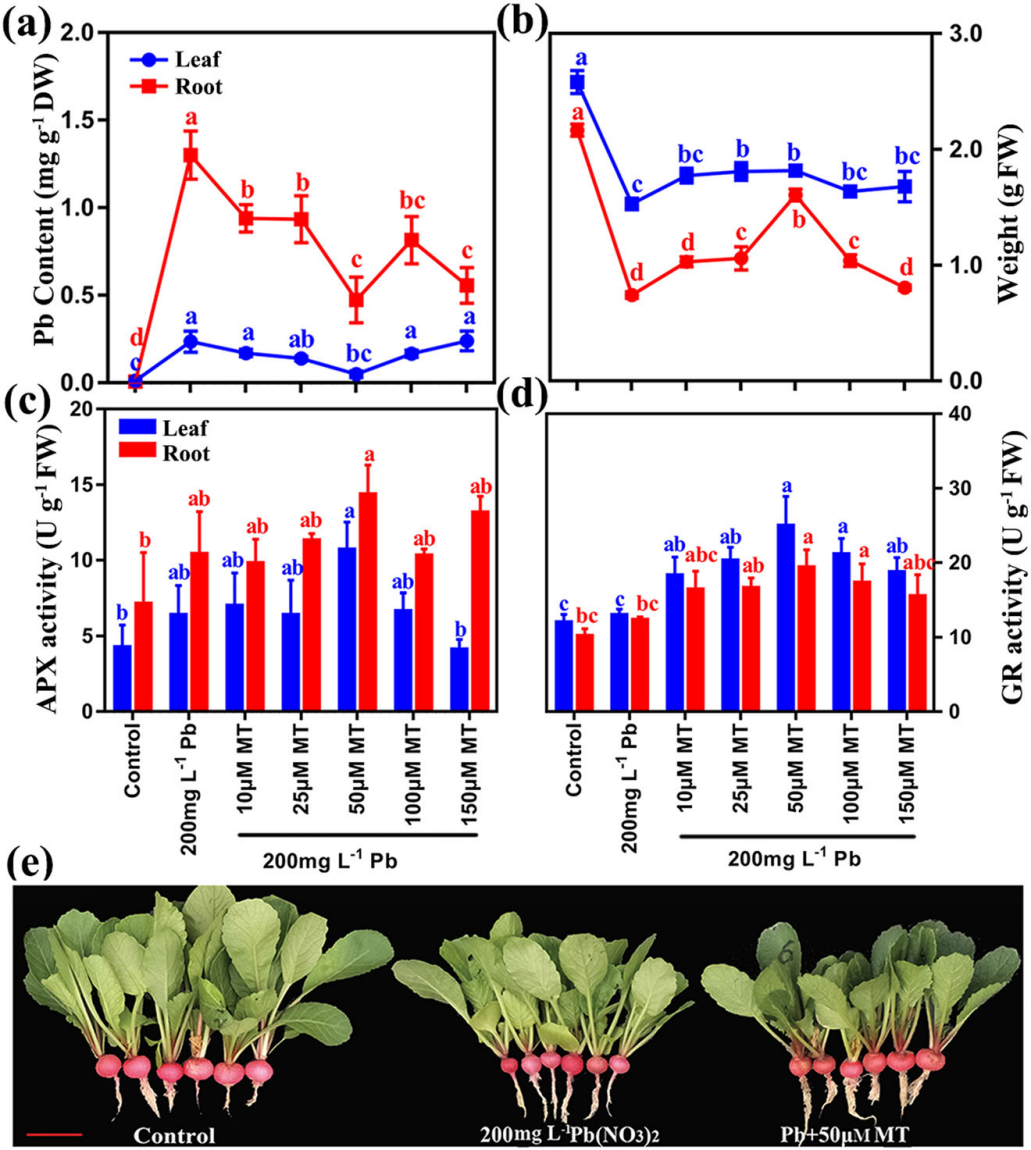
Effects of MT treatment on Pb stress in radish. **a** Changes in Pb contents (mg g^−1^ DW), DW: dry weight. **b** Weight of roots and leaves under different concentrations of MT treatment, FW: fresh weight. **c**, **d** Melatonin-induced changes in the activity of ascorbate peroxidase (APX) and glutathione reductase (GR). **e** Growth conditions of radish under Pb-free (Control), Pb stress (Pb200), and 50 μM MT treatment under Pb stress (Pb_50MT). Columns with different letters indicate significant differences at *P* < 0.05 according to Duncan’s multiple range test

To characterize the roles of MT under Pb stress, the Pb contents and antioxidant enzymes of radish roots and leaves treated with Pb(NO_3_)_2_ (200 mg L^−1^) (Pb200) or Pb200 plus MT (0, 10, 25, 50, 100 and 150 μM) were assayed (Fig. 1). The Pb contents of both leaves and roots were significantly decreased after MT treatment, especially in Pb200 plus 50 μM MT (Pb_50MT) (Fig. 1a). However, the Pb content under the treatments with different MT concentrations was higher than that under the Pb-free treatment (Control). Moreover, the weights of the roots and leaves under Pb_50MT were larger than those under Pb200, and the activities of antioxidant enzymes including ascorbate peroxidase (APX) and glutathione reductase (GR) were increased under Pb stress at different melatonin concentrations, both of which reached the maximum level at 50 µM melatonin, indicating that MT treatment relieved the toxicity of Pb stress in both the leaves and roots of radish plants (Fig. 1b–e).

### Features of the radish DNA methylome under Pb and MT treatment

**Fig. 2:**
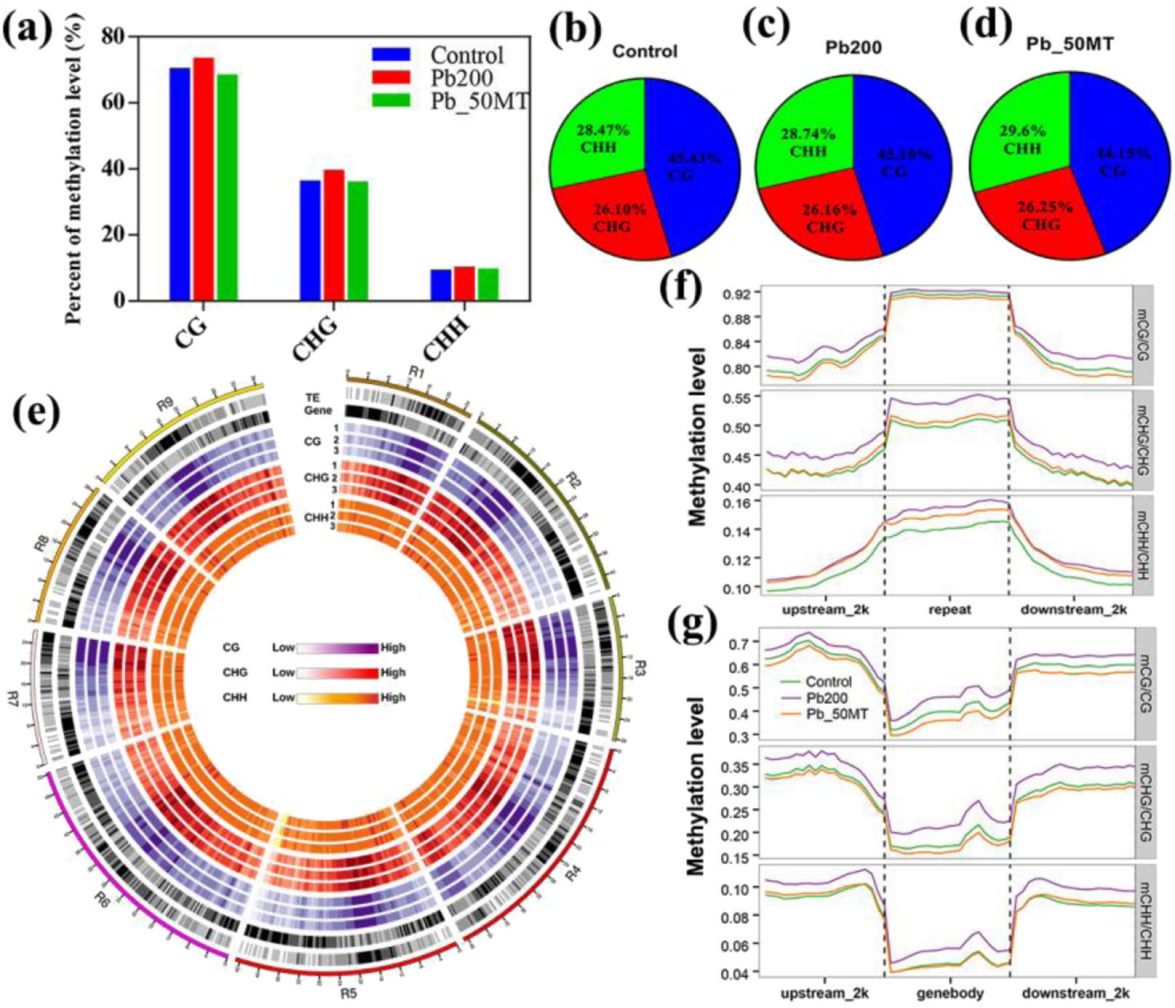
Radish single-base resolution maps and features of DNA methylation. **a** Global levels of radish DNA methylation in three contexts. **b**–**d** Relative proportions of mCs in three sequence contexts (CG, CHG, and CHH) in radish, respectively. **e** The landscape of DNA methylation in nine chromosomes of radish. From outer to inner: TE density, gene density and methylation of CG, CHG and CHH (1: Control, 2: Pb200, 3: Pb_50MT). Black indicates high gene/TE density. **f** DNA methylation patterns of the gene body and flanking regions in the CG, CHG and CHH sequence contexts. **g** DNA methylation patterns of repeat and flanking regions in the CG, CHG and CHH sequence contexts. Upstream_2 kb indicates the 2000 bp upstream from transcriptional start sites (TSSs), and downstream_2 kb indicates the 2000 bp downstream from transcriptional end sites (TESs)

To explore the roles of MT in the reduction of the Pb content, the dynamic patterns of DNA methylation among the three treatments (Control, Pb200 and Pb_50MT) were characterized by whole-genome bisulfite sequencing (WGBS) (Supplementary Table S1). The percentage of methylated cytosines (mCs) showed that the methylation level increased under Pb treatment, and a subsequent slight decrease was observed under MT treatment. The average genome-wide methylation level of Control was 70.47%, 36.44% and 9.4% in the CG, CHG and CHH contexts, respectively. Compared with that under Pb200, the methylation level in CG, CHG and CHH under Pb_50 MT was reduced by ~5.09%, 3.55% and 0.51%, respectively (Fig. 2a, Supplementary Table S1). Among the Control, Pb200 and Pb_50MT libraries, the proportion of mCs in the CG context (~45.43%, 45.10% and 44.14%) was much higher than that in CHG (~26.10%, 26.16% and 26.25%) and CHH (~28.47%, 28.74% and 29.60%) (Fig. 2b-d).

From the chromosome-level perspective, heterochromatic regions with a high density of transposable elements (TEs) were strongly methylated, which revealed that the hypermethylation level in repeat regions might be responsible for the repression of active transposons. However, a reduced methylation level was characterized in gene-rich euchromatic regions under all three treatments (Figs. 2e and S1). The methylation levels of each sequence context were high at the repeat body region and rapidly decreased in the flanking 2 kb regions, whereas the gene body region exhibited lower methylation levels than upstream and downstream regions, which was in accordance with findings in *Arabidopsis*^27^ (Fig. 2f, g).

### MT treatment decreases DNA methylation under Pb stress

**Fig. 3:**
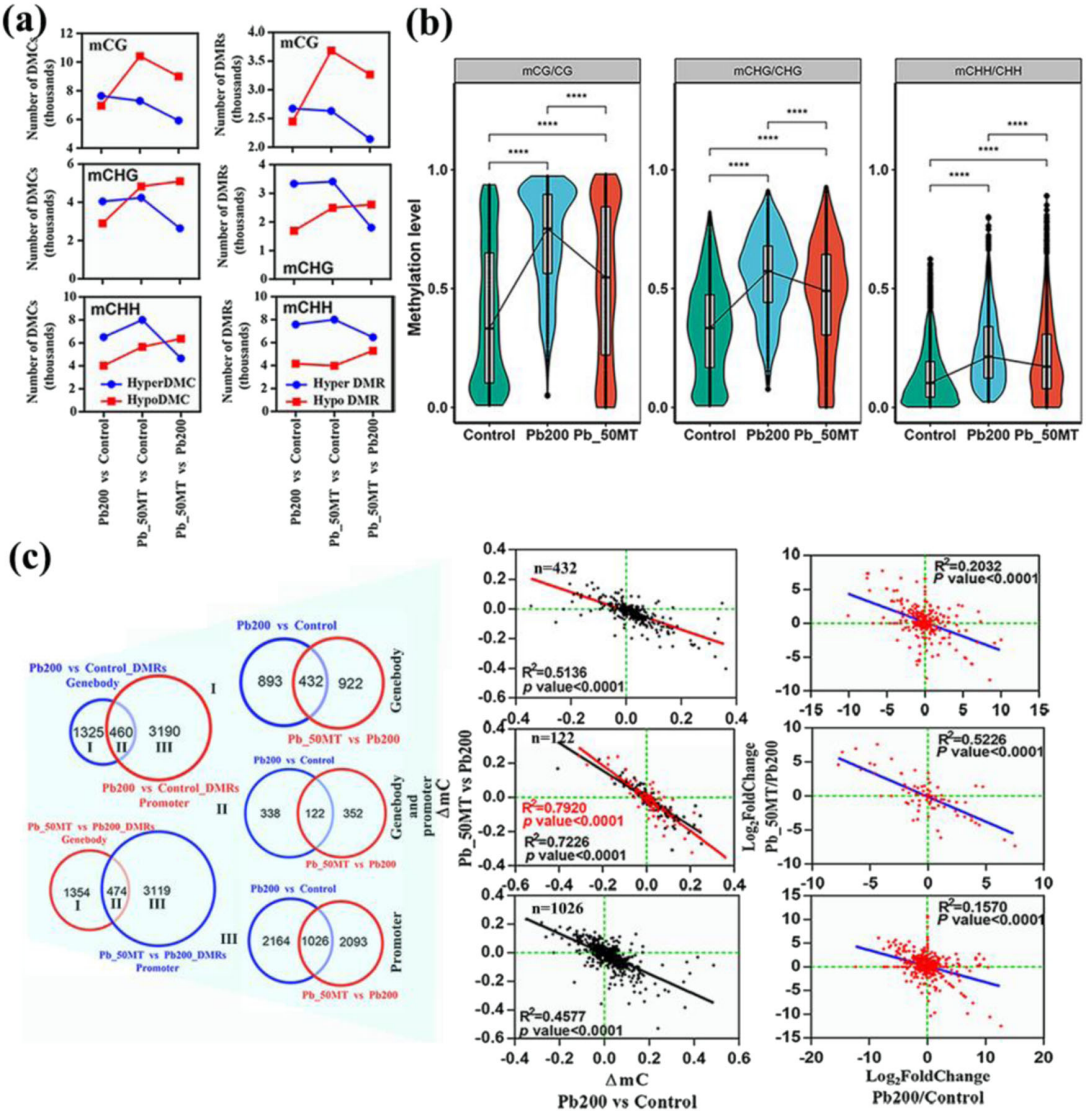
MT-induced demethylation under Pb stress in radish. **a** Numbers of DMCs and DMRs in Pb200 vs Control, Pb_50MT vs Control, and Pb_50MT vs Pb200 are shown for the mCG, mCHG, and mCHH sequence contexts. **b** The DNA methylation distribution of Pb200 vs Control hyper-DMRs in Pb_50MT for the mCG, mCHG and mCHH sequence contexts. **c** The correlation of differential DNA methylation and expression levels of overlapping DMR-associated genes between Pb200 vs Control and Pb_50MT vs Pb200. Statistical significance was determined by paired two-tailed Student’s *t* test, *P < 0.05; **P < 0.01; ***P < 0.001; ns not significant

As shown in Fig. 2, the genomic methylation level under Pb200 was higher than that under Control and Pb_50MT. Hence, the differentially methylated cytosines (DMCs) and regions (DMRs) among the three treatments were analyzed to characterize the variations in methylation levels. In total, 11930, 11485 and 13100 DMRs were identified in Pb200 vs Control, Pb_50MT vs Pb200 and Pb_50MT vs Control by comparing the methylomes, respectively. In the Pb200 vs Control group, the number of hyper-DMCs was larger than that of hypo-DMCs, similar to the number of DMRs in all three contexts. However, there were more hypo-DMC and hypo-DMR numbers in the CG and CHG contexts in Pb_50MT vs Pb200 (Fig. 3a). To explore the potential roles of MT treatment in DNA demethylation under Pb stress, Pb200 vs Control hyper-DMRs and Pb_50MT vs Pb200 hypo-DMRs were used for further analysis. The methylation level of most Pb200 vs Control hyper-DMRs was lower in Pb_50MT than in Pb200, and the DNA methylation level of hypo-DMRs between Pb_50MT and Pb200 was lower in Control than in Pb200, indicating that the increased hypermethylation level under Pb stress might change in favor of hypomethylation after MT treatment (Figs. 3b and S2a–c). Furthermore, 1028 (34.6%) hyper-DMR-associated genes of Pb200 vs Control overlapped with hypo-DMR-associated genes of Pb_50MT vs Pb200 (Figs. S2d and S3a). The differential expression level of all DMR-associated genes in Pb200 vs Control was negatively related to that in Pb_50MT vs Pb200, and the overlapping DMR-associated genes exhibited a higher correlation (Fig. S3b).

Based on the number of hyper/hypo-DMRs, the genomic distribution of hyper and hypo-DMRs showed that DMRs of the CG context were mainly enriched at promoter and exon regions among all three groups, while the proportion of exons was decreased with increasing repeats in both mCHG and mCHH DMRs. In particular, hyper-DMRs of mCHG and mCHH were enriched at promoter and repeat regions (Fig. S3c). To investigate the dynamic changes in methylation levels in promoter and gene body regions, differential methylation analysis of Pb200 vs Control and Pb_50MT vs Pb200 was performed. In total, 1785 and 3650 differentially methylated genes associated with gene body and promoter regions were obtained in Pb200 vs Control, while 1828 and 3593 genes harboring DMRs were identified in Pb_50MT vs Pb200 at the gene body and promoter regions, respectively. These DMR-associated genes were classified into three classes according to their location, in which the differential methylation and expression levels of these genes between two groups were negatively correlated. Moreover, the correlation coefficient showed that MT may change gene expression by altering the methylation levels of promoter and gene body regions together (Fig. 3c).

### Correlation between DNA methylation and gene expression under Pb stress

**Fig. 4:**
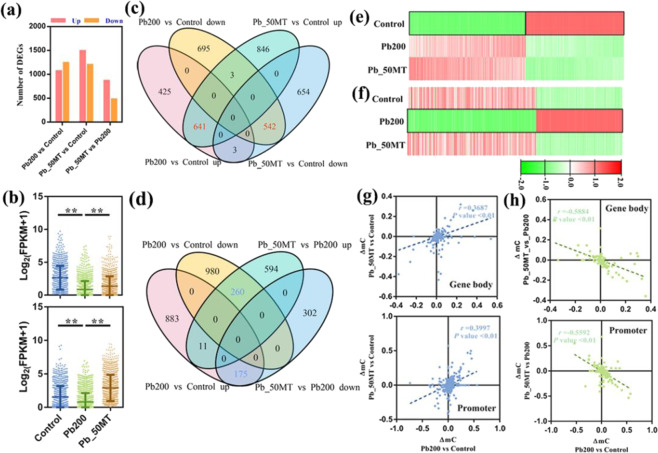
Expression and methylation patterns of MT-induced DEGs. **a** The numbers of up- and downregulated DEGs in Pb200 vs Control, Pb_50MT vs Control, and Pb_50MT vs Pb200. **b** The expression patterns of Pb200 vs Control downregulated and Pb_50MT vs Pb200 upregulated DEGs. **c** The overlapping DEGs between Pb200 vs Control and Pb_50MT vs Control. **d** The overlapping DEGs between Pb200 vs Control and Pb_50MT vs Pb200. **e**, **f** The expression patterns of overlapped DEGs. **g**, **h** Correlation of differential methylation levels of overlapping DEGs

To further investigate whether MT-induced DNA demethylation is associated with changes in gene expression, transcriptome profiles of three treatments and differential expression analysis were performed. In all, 2309 (1069 up- and 1240 downregulated), 2689 (1490 up- and 1199 downregulated), and 1342 (865 up- and 477 downregulated) differentially expressed genes (DEGs) were obtained for Pb200 vs Control, Pb_50MT vs Control, and Pb_50MT vs Pb200, respectively (Fig. 4a). The expression analysis of downregulated DEGs in Pb200 vs Control and upregulated DEGs in Pb_50MT vs Pb200 indicated that most genes with low expression in Pb200 showed an upregulated expression pattern in Control and Pb_50MT (Fig. 4b). Interestingly, most of the shared DEGs between Pb200 vs Control and Pb_50MT vs Control showed a similar expression trend, suggesting that these DEGs may play key roles in the biological process of the Pb stress response (Fig. 4c). However, a majority of overlapping DEGs between Pb200 vs Control and Pb_50MT vs Pb200 that exhibited distinct expression patterns may be regulated by MT to reduce Pb toxicity (Fig. 4d). In addition, the differential methylation level of shared DEGs between Pb200 vs Control and Pb_50MT vs Control exhibited a significantly positive correlation, while the overlapping DEGs between Pb200 vs Control and Pb_50MT vs Pb200 showed a markedly negative correlation in differential methylation levels, which were consistent with the changes in gene expression (Fig. 4e–h).

**Fig. 5:**
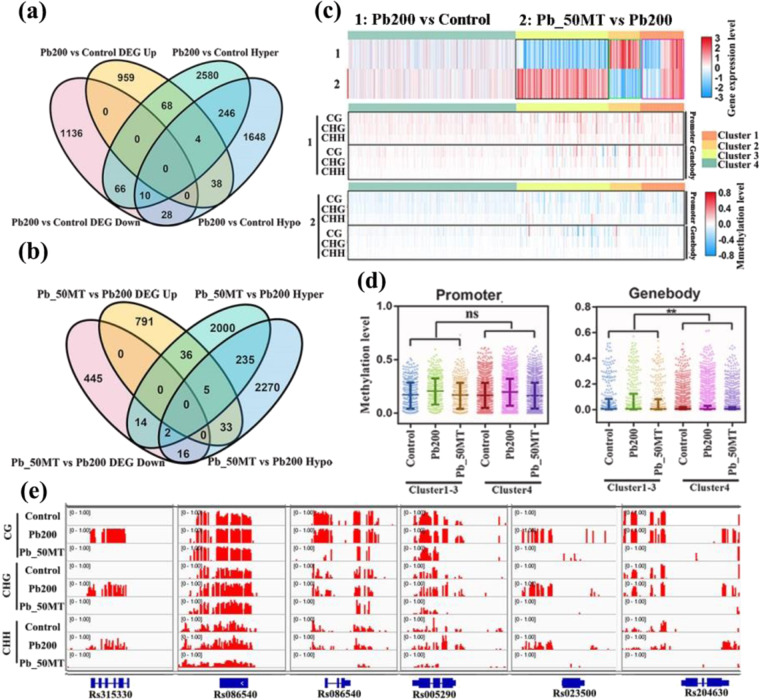
The expression and methylation changes in DMR-associated genes after Pb(NO_3_)_2_ and MT treatment in radish. **a**, **b** The overlap between DMR-associated genes and differentially expressed genes (DEGs) in (**a**) Pb200 vs Control and (**b**) Pb_50MT vs Pb200. **c** Cluster of expression and methylation of Pb200 vs Control hyper-DMR-associated genes and Pb_50MT vs Pb200 hypo-DMR-associated genes. **d** Comparison of methylation levels between Clusters 1-3 and Cluster 4 in promoters and gene bodies. **e** IGV screenshots of six representative hyper and hypomethylated genes in the Control, Pb200, and Pb_50MT groups

Additionally, differentially expressed and methylated region-associated genes (DEGs and DMR-associated genes) overlapped and were used to explore the potential expression levels of MT-induced methylation changes associated with Pb stress. A total of 76 hyper-DMR- and 38 hypo-DMR-associated genes were significantly downregulated, and 72 hyper-DMR- and 42 hypo-DMR-associated genes showed significantly upregulated expression in Pb200 vs Control (Fig. 5a). Similarly, 16 downregulated and 41 upregulated DEGs were hypermethylated, while 18 downregulated and 38 upregulated DEGs were hypomethylated in Pb_50MT vs Pb200 (Fig. 5b). These results suggested that the methylation changes partially affected the transcriptional alterations of several DEGs with no differential transcript abundance.

Furthermore, the expression levels of all Pb200 vs Control hyper-DMR-associated genes and Pb_50MT vs Pb200 hypo-DMR-associated genes were analyzed because MT mainly decreased the methylation level (Figs. S2d and S3a). Among 4506 DMR-associated genes, 2003 were expressed in at least one sample (FPKM > 1). The expression change was analyzed for each gene in these two groups (Log_2_Pb200/Control and Log_2_Pb_50MT/Pb200), and all genes were assigned into four clusters as follows: cluster 1: 74 genes with similar expression changes in two groups, cluster 2: 53 genes that were upregulated in Pb200 vs Control and downregulated in Pb_50MT vs Pb200, cluster 3: 155 genes that were downregulated in Pb200 vs Control and upregulated in Pb_50MT vs Pb200, and cluster 4: 1721 genes that were not significantly changed (Fig. 5c). Interestingly, the methylation levels of cluster 1–3 genes exhibited significant differences in gene bodies but not in promoters compared with genes in cluster 4, indicating that the changes in DNA methylation in the gene body were insufficient to lead to alterations in gene expression among these genes in cluster 4 (Fig. 5d). As shown in Fig. 5e, visualization of the methylation level for DMR-associated genes in clusters 1-4 was performed. The methylation change of an HM transport detoxification domain-containing protein (Rs204630), which was hypermethylated in Pb200 compared with the Control and Pb_50MT groups, are also presented. These results supported a critical role of MT in DNA demethylation with changes in gene expression under Pb stress in radish.

### Functional annotation of DEGs and methylation analysis of HM-associated genes

**Fig. 6:**
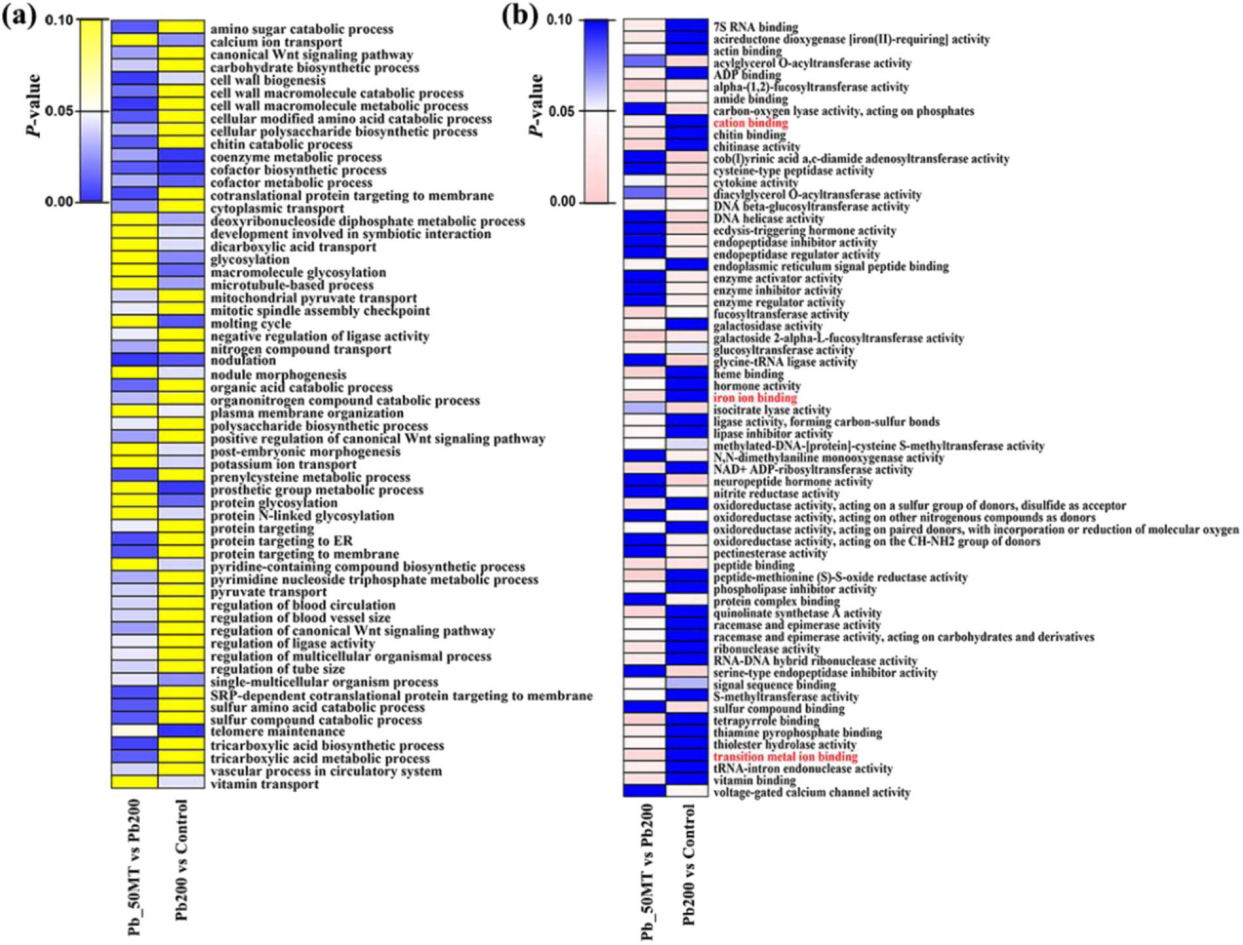
GO enrichment terms of DMR-associated genes in Pb200 vs Control and Pb_50MT vs Pb200. **a** GO biological process terms enriched in DMR-associated genes of Pb200 vs Control and Pb_50MT vs Pb200. **b** GO molecular function terms enriched in DMR-associated genes of Pb200 vs Control and Pb_50MT vs Pb200

To understand the potential role of MT-induced differential expression and DNA demethylation, Gene Ontology (GO) analysis was performed. The GO analysis of DMR-associated genes showed that cell well biogenesis, coenzyme metabolic process and single-multicellular organism process in the biological process category as well as peptide binding and amide binding in the function category were significantly enriched terms in both Pb200 vs Control and Pb_50MT vs Pb200. However, the majority of GO terms, including calcium ion transport, vitamin transport and sulfur compound binding, were unique in Pb200 vs Control (Fig. 6a). In addition, uniquely enriched GO terms associated with ion binding were found in Pb_50MT vs Pb200, such as cation binding, iron ion binding and transition metal ion binding (Fig. 6b). These ion binding-associated genes (promoter and gene body regions) were mainly hypermethylated in Pb200 vs Control, while the majority of them were hypomethylated in Pb_50MT vs Pb200 (Fig. S4a). Similarly, most of the downregulated genes in Pb200 vs Control showed upregulated expression in Pb_50MT vs Pb200 (Fig. S4b). In addition, the significantly enriched GO terms of DEGs in the three comparisons were further investigated. Both Pb200 vs Control downregulated DEGs and Pb_50MT vs Pb200 upregulated DEGs were significantly enriched in response to oxidative stress, oxidation–reduction process and antioxidant activity (corrected *p*-value < 0.05), suggesting that these genes might be involved in MT-induced Pb detoxification by enhancing antioxidant enzyme activity (Fig. S5). Furthermore, downregulated DEGs of Pb_50MT vs Control, consisting of 16 differentially expressed multidrug and toxic compound extrusion (MATE) genes, were mainly enriched in oxidoreductase activity and drug transporter activity.

**Fig. 7:**
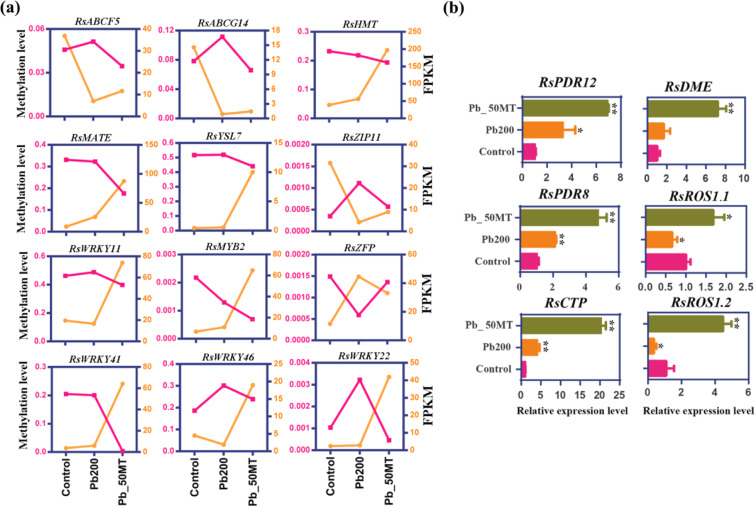
Expression and methylation analysis of HM-associated genes in response to Pb stress. **a** The expression and DNA methylation levels of HM-associated genes and TFs. **b** The expression levels of Pb-associated and demethylase genes under different treatments by RT-qPCR. Statistical significance was determined by paired two-tailed Student’s *t* test, *P < 0.05; **P < 0.01; ***P < 0.001; ns: not significant

A total of 63 DEGs associated with metal transport were identified to be involved in Pb reduction and detoxification. Eight out of 12 ABC transporters showed higher expression levels in Control than in Pb_50MT, while 12 out of 16 MATE efflux proteins were upregulated under MT treatment. Additionally, 23 and 17 DEGs, including ABC transporter, heavy metal transport protein and MATE efflux protein, were upregulated with DNA methylation changes in Pb_50MT vs Pb200 and Pb200 vs Control, respectively (Figs. S6 and 7a). Interestingly, differentially methylated TFs involved in HM stresses were identified in this study, and the number of MYB and WRKY TFs accounted for a large proportion of these, which may further affect the expression of metal transporter genes (Figs. 7a and S7a). RT-qPCR analysis showed that *RsPDR8*, *RsPDR12* and *RsCTP* were obviously upregulated under Pb and MT treatment. Moreover, the *RsDME* and *RsROS1* genes, which can prevent DNA methylation, were significantly upregulated in Pb_50MT compared with Pb200, indicating that they may have a large effect on MT-induced DNA demethylation (Fig. 7b). These results suggested that MT-induced DNA demethylation, with the involvement of *RsROS1* and *RsDME*, might alter the expression of HM-associated genes and TFs and lead to Pb reduction and detoxification.

## Discussion

Lead (Pb), one of the most widespread and dangerous metal pollutants, presents high toxicity to plants and causes serious health problems throughout the food chain^2^. Melatonin (MT) has been proven to play a critical role in enhancing plant tolerance to HM stresses^15,28^. Radish, as a major root vegetable crop worldwide, has been demonstrated to be important in studying root HM accumulation and tolerance in root and tuberous crops^29^. However, systematic investigation of the DNA methylation level has not been performed in radish, especially under the application of MT. In this study, the influence of Pb stress and MT treatment on the association between DNA methylation and expression levels was explored with WGBS and RNA-seq technology. To our knowledge, this is the first study to investigate and characterize root methylome changes and the role of MT in decreasing Pb toxicity in radish.

### MT-mediated regulation of signal transduction and activation of antioxidant systems

Exogenous MT treatment enhances Cd immobilization in the cell wall and vacuoles and minimizes Cd toxicity^30^. In tomato, the Cd concentration in leaves was reduced by supplementation with exogenous MT^31^. Similarly, our results showed that exogenous MT application reduced Pb^2+^ accumulation and increased root and leaf weight (Fig. 1a, b). Diverse calcium-sensing proteins, including calmodulins (CaM), calcineurin B-like proteins (CBLs), CaM-like proteins (CML) and calcium-dependent protein kinases (CDPK), were found to be involved in heavy metal (HM) uptake, accumulation and detoxification^32,33^. It was reported that ROS and CDPK played a role in Pb^2+^-induced cell death with the involvement of Ca^2+^ and triggering of mitogen-activated protein kinase (MAPK) activity^33,34^. In this study, 12 DEGs, encoding CaM (3), CBL (3), CML (3), CPK (1) and CDPK (2), were significantly upregulated under Pb stress with MT treatment. Moreover, six upregulated genes encoding MAPK and MAPKKK were found under Pb or Pb with MT treatment (Fig. S7b). These results revealed that calcium-dependent and MAPK signal transduction might be activated under Pb stress or Pb stress with MT treatment, which would further affect the activation of antioxidant systems and the expression of TFs and HM-responsive genes.

Previous studies revealed that MT acted as an antioxidant combating ROS accumulation under various abiotic stresses^21,35,36^. In the melon response to copper stress, MT treatment increased antioxidant enzyme activities and promoted root development^35^. In rice shoots, MT enhanced stress tolerance through ROS scavenging and transporters caused by changes in global gene expression^21^. In wheat, the MT treatment increased antioxidants and enhanced Al exclusion from root tips^36^. In our study, the antioxidant enzyme activities of APX and GR were increased in radish roots and leaves under MT treatments, which was consistent with the findings in wheat and tomato^31,36^ (Fig. 11c, d). Additionally, several antioxidant DEGs were markedly enriched in antioxidant activity (Fig. S5b). In detail, *ascorbate peroxidase 2* (*APX2*) and eight of 21 *peroxidases* (*POD52* and *POD64*) were obviously upregulated under MT treatment, which promoted radish plants to increase antioxidant enzyme activities to cope with Pb stress. Many studies have shown that glutathione S-transferases (GSTs) can accelerate HM-induced ROS scavenging by catalyzing the conjugation of GSH with HM ions into low-toxicity complexes, which are transported into the vacuole to reduce HM toxicity^37^. In this study, the expression of eight DEGs encoding *GST* was increased under MT treatment (Fig. S8). In contrast, most of these differentially expressed antioxidant genes exhibited decreasing methylation levels. Together, these findings indicated that exogenous MT can alleviate Pb-induced oxidative stress and achieve Pb^2+^ detoxification partially by regulating several redox and antioxidant genes and activating specific antioxidant systems in radish roots.

### MT-induced reduction of Pb accumulation was accompanied by methylation alteration in radish

Currently, WGBS has been extensively used to decode single-base genome-wide methylation in plants, including *Arabidopsis*^27^, tea^38^, and apple^13^. In this study, global DNA methylation patterns were characterized through bisulfite sequencing, and the first single-base resolution DNA methylation map of radish was produced. In rice in response to Cd stress, the methylation level of genes was changed, followed by altered expression levels, and 5-azacytidine promoted growth and Cd accumulation by enhancing the transcription of genes involved in HM transport^12^. Exposure to Cd elevated DNA methylation at the genome-wide level in *Arabidopsis* roots^39^. In the present study, the genome-wide DNA methylation level was increased under Pb stress and obviously reduced after 50 μM MT treatment (Fig. 2). Interestingly, the methylation level of most hyper-DMRs in Pb200 vs Control was lower in Pb_50MT than in Pb200, while most hypo-DMRs between Pb_50MT and Pb200 exhibited higher methylation levels in Pb200 than in Control (Figs. 3a, b and S3a–c). These results agreed with those found in grape berries, which showed that exogenous melatonin treatment decreased the methylation levels of various gene regions in the CHG and CHH contexts^40^. It was reported that the DNA demethylase *DML2* was downregulated under cold stress in tomato, which resulted in the hypermethylation of the promoter and silencing of genes involved in the biosynthesis of flavor volatiles^41^. Moreover, Cd-induced lower expression of three DNA demethylase genes (*ROS1*, *DML2* and *DML3*) resulted in higher DNA methylation in *Arabidopsis*^39^. In this study, RT-qPCR analysis of DNA demethylase genes showed that *RsROS1* was downregulated in Pb200 and upregulated in Pb_50MT, while *RsDME* presented higher expression levels in Pb200 than in Control, indicating that these genes may play a major role in decreasing the methylation level after MT treatment (Fig. 7b).

Generally, a high methylation level could inhibit gene expression, especially in the upstream regions of genes, and highly methylated genes exhibited lower expression levels^38^. In total, 76 and 72 hyper-DMR-associated genes were significantly down- and upregulated in Pb200 vs Control, respectively, indicating that DNA methylation played a vital role in suppressing expression of specific genes (Fig. 5a, b). In plants, the ion-exchangeable sites located in cell walls can be bound by Pb^2+^ to precipitate extracellularly^42^. Hence, the metal transporter genes associated with ion binding might be responsible for metal ion uptake, translocation and detoxification. In our study, uniquely enriched GO terms, including cation binding, iron ion binding and transition metal ions, were found in Pb_50MT vs Pb200, and the majority of ion binding-associated genes showing higher methylation levels in Pb200 were hypomethylated in Pb_50MT vs Pb200, suggesting that the alteration of DNA methylation by MT may play a critical role in plant response to Pb stress (Figs. 6b and S4).

### MT-mediated regulatory network of Pb accumulation and detoxification

To reduce the accumulation of HMs, plants have adopted several effective strategies to restrict HM ions to the cell by binding them to the cell wall or excluding them^43–45^. Previous evidence has shown that several metal transporter genes, such as ATP-binding cassette (ABC), zinc/iron transport protein (ZIP), multidrug and toxic compound extrusion (MATE) and HM transport protein (HMT) genes, are involved in activating the efflux of HM ions across the plasma membrane or sequestering HM into vacuoles to avoid toxicity^12,46,47^. The DNA methylation level has been considered a ‘silencing’ epigenetic mark and vital regulator of plant growth and stress responses through the trigger of transcriptional and posttranscriptional control of gene expression^9^. Previous studies showed that promoter methylation inhibited gene expression in the hypermethylated gene region^48,49^. As shown in Fig. 7a, the methylation level of several metal transporter genes, including ABC transporters (*RsABCF5* and *RsABCG14*), *RsYSL7*, and *RsZIP11*, was decreased, while their corresponding expression level was upregulated under MT treatment (Figs. 7a and S6a). Moreover, RT-qPCR analysis showed that both the *PLEIOTROPIC DRUG RESISTANCE TRANSPORTER12* (*RsPDR12*) and *RsPDR8* genes were significantly upregulated under Pb_50MT. In *Arabidopsis*, *AtPDR12* acts as a pump of the plasma membrane to exclude Pb^2+^ or Pb^2+^-containing toxic compounds from the cytoplasm^50^. *AtPDR8* was demonstrated to exclude Pb from epidermal cells and enhance Pb resistance in *Arabidopsis*^51^. These results indicated that the differential expression of these specific low-methylated metal transporter genes might contribute to reducing MT-induced Pb accumulation in radish plants.

It has been reported that several TFs, including MYB, WRKY, and zinc-finger domain-containing protein (ZFP) genes, are induced under HM stresses^44,52^. *AtWRKY13* directly activates the expression of the *AtPDR8* gene to reduce Cd accumulation^44^. In addition, *AtMYB49* directly regulates the expression of the *AtbHLH38*, *AtbHLH101*, *AtHIPP22* and *AtHIPP44* genes to positively control Cd accumulation in *Arabidopsis*^52^. In this study, *RsMYB2* and four *RsWRKY* family genes (*RsWRKY11*, *22*, *44* and *46*) presented lower methylation levels with increased expression under MT treatment (Fig. 7a). It is reasonable to conclude that MT-induced DNA demethylation contribute to reducing Pb accumulation by altering the expression of several specific HM transporter genes in radish (Figs. 7, S4, S7a and 8).

**Fig. 8:**
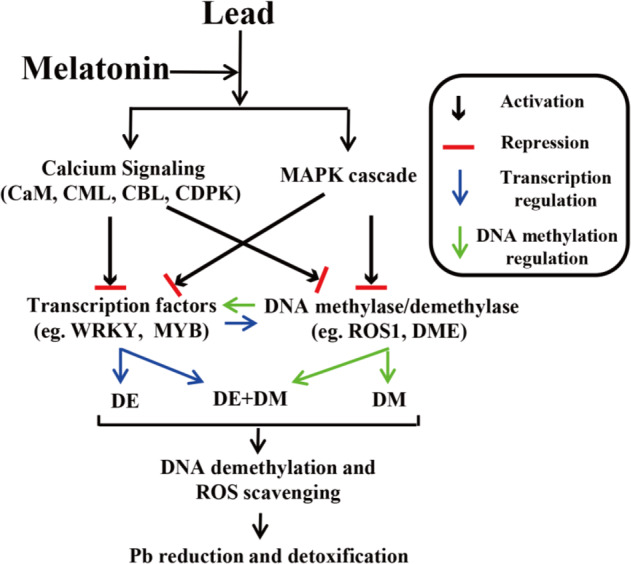
A hypothetical model of MT-induced Pb reduction and detoxification in radish. CaM calmodulin, CML calmodulin-like protein, CBL calcineurin B-like protein, CDPK calcium-dependent protein kinase, MAPK mitogen-activated protein kinase, ROS1 REPRESSOR OF SILENCING 1, DME DEMETER, DE differential expression, DM differential methylation, ROS reactive oxygen species

## Conclusion

In summary, a single-base resolution Pb-induced DNA methylation map was first constructed, and the role of MT-induced DNA demethylation was identified in radish. Antioxidant genes, including *RsAPX2* and *RsPOD64*, which showed mostly low methylation levels, were upregulated to enhance antioxidant enzyme activities. Notably, MT-induced DMR-associated genes enriched in metal ion binding and oxidation–reduction process terms might play a critical role in the MT-mediated regulatory network of Pb accumulation and detoxification in radish. The methylation levels of TFs, including WRKYs and MYBs, which regulate metal transporter genes, including *RsPDR12*, could be changed by MT treatment. Moreover, MT-induced demethylation of several HM-responsive genes and TFs could further promote the process of Pb detoxification by regulating transcription levels in radish. Further functional characterization of these DEGs and their methylation features would be useful for better elucidation of the molecular mechanism underlying Pb stress responses in radish. These findings provide fundamental insights for further investigation on Pb accumulation and detoxification in root vegetable crops.

## Materials and methods

### Plant materials and growth conditions

Radish seeds of the advanced inbred line ‘NAU-YH’ were surface-sterilized, rinsed, and germinated for 3 days and then cultivated in a controlled growth chamber under a photoperiod of 14 h light at 25 °C and 10 h dark at 18 °C for three weeks. Seedlings of similar sizes were transferred into a plastic container with half-strength Hoagland nutrient solution. After 5 days, plants were treated with 0 and 200 mg L^−1^ Pb(NO_3_)_2_. Hydroponic solution was refreshed every five days. Seedlings grown in Pb-free nutrient solution were used as controls. To determine the optimal dose of melatonin (MT) that could effectively decrease accumulation, the foliar portion of Pb-treated radish seedlings was sprayed with different concentrations of MT (0, 10, 25, 50, 100 and 150 μM) at 8:00 pm. Based on the preliminary dose trial, 50 μM MT was selected for the rest of the experiments, considering its effect in reducing Pb contents. Three replicates were used for each treatment, with 6–8 plants for each replicate.

### Determination of antioxidant enzyme activities and Pb contents

To measure the antioxidant enzyme activities, 0.1 g of fresh leaf and root samples was suspended in potassium phosphate buffer (50 mmol L^−1^, pH 7.0) containing 0.1 mmol L^−1^ EDTA and 1% polyvinylpyrrolidone (w/v). The fully vortexed homogenate was centrifuged for 15 min at 12,000 × *g* (4 °C), and the supernatant was collected to detect the activities of glutathione reductase (GR) and ascorbate peroxidase (APX) enzymes according to previous studies^53^. For the Pb content, roots and leaves were separately collected and weighed, dried at 85 °C for 24 h, and ground into powder with a mortar. Each sample (0.2 g) was transferred into a high-pressure polytetrafluoroethylene vessel to which 5 mL HNO_3_ was added. The vessel was sealed with a screwcap and digested by a microwave (Mars 6, CEM Technologies, USA). The digested solutions were used to determine the Pb content by an inductively coupled plasma optical emission spectrometer^54^ (ICP-OES, Thermo Fisher iCAP 7400).

### Construction of genomic methyl cytosine libraries and methyl-Seq analysis

Total genomic DNA was extracted from the roots of seedlings, including Pb-free (Control), Pb-treated (Pb200), and Pb plus 50 μM MT-treated (Pb_50MT) seedlings, using a Plant Genomic DNA Kit (Tiangen, Beijing, China) for whole-genome Methyl-Seq. DNA quality was checked using a NanoPhotometer^®^ spectrophotometer (IMPLEN, CA, USA). A total of 5.2 µg genomic DNA was fragmented by sonication to 200–300 bp with Covaris S220, followed by end repair and adenylation. These DNA fragments were treated twice with bisulfite using an EZ DNA Methylation-GoldTM Kit (Zymo Research). The prepared library was sequenced on the Illumina HiSeq platform (Novogene, Beijing, China), and base calling was performed with the Illumina CASAVA pipeline. FastQC (fastqc_v0.11.5) was used to perform basic statistics on the quality of raw reads. Clean reads were generated by filtering out adaptor sequences, contaminants, and low-quality reads using fastp software^55^ with the following parameter settings: (1) –length_required = 36; (2) –cut_front_window_size = 1; (3) –cut_front_mean_quality = 3; (4) –cut_tail_window_size = 1; and (5) –cut_tail_mean_quality = 3. Bismark software (version 0.16.3) was used to perform alignments of bisulfite-treated reads to a reference genome^56,57^.

### Methylation level and differentially methylated region (DMR) analyses

To calculate the methylation levels of the sequences, a sequence was divided into multiple bins, and the bin size was 10 kb. A sliding-window approach was applied for methylation-level analysis. With window size = 3000 bp and step size = 600 bp, and the sum of methylated and unmethylated read counts was calculated in each window. The methylation level (ML) for each window or C site showed the fraction of methylated C and was defined as ML(C) = reads(mC)/[reads(mC)+reads(C)]^58^.

The Bioconductor package DSS^59^ (Dispersion Shrinkage for Sequencing) was used to identify differentially methylated regions (DMRs) following default parameter settings with a reduced smoothing size (smoothing span = 200). According to the distribution of DMRs throughout the genome, genes related to DMRs were defined as DMR-associated genes whose gene body region (from TSS to TES) or promoter region (upstream 2 kb from the TSS) overlapped with DMRs.

### RNA sequencing and differential analysis

A total of 3 μg RNA per sample (Control, Pb200 and Pb_50MT) was used as an input material for RNA sample preparation. The libraries were sequenced on an Illumina HiSeq 2500 platform, and 150 bp paired-end reads were generated. Paired-end clean reads were aligned to the reference genome^56^ using Bowtie and TopHat 2 programs^60,61^. The mapped reads of each sample were assembled by StringTie (v1.3.1) in a reference-based approach^62^. The gene expression levels of mRNA reads were calculated using the fragments per kilobase of transcript per million fragments mapped (FPKM) approach, and DESeq2 was used to obtain differentially expressed genes (DEGs) with fold change >1 and *P*-value < 0.05.

### RT-qPCR analysis

Total RNA was isolated from the control and treated radish roots using an RNA simple total RNA kit (Tiangen, Beijing, China). RNA was reverse transcribed into cDNA using a PrimeScript™ II 1st Strand cDNA Synthesis Kit (Takara, Dalian, China). RT-qPCR analysis was conducted using SYBR Green PCR Master Mix with ROX (Takara, Dalian, China). Each 20 μl reaction contained 10 μl of 2×SYBR Green PCR Master Mix (Takara, Dalian, China), 0.2 μM of each primer, and 2 μL of diluted cDNA. PCR was performed on a LightCycler^®^ 480 System (Roche, Mannheim, Germany) with the following thermal cycling conditions: 95 °C for 3 min and 40 cycles of 95 °C for 5 s, 58 °C for 30 s, and 72 °C for 10 s^63^. The $2^{-\Delta\Delta{{C}_{\rm{T}}}}$ method was used to calculate relative expression levels^64^. Three biological and technical replicates were performed, and the *RsActin* gene was employed as the internal standard^57^. The significance of differences between groups was evaluated using Student’s *t* test. Analyses were performed with GraphPad Prism software (GraphPad Software, San Diego, California).

## Supplementary information

Supplementary data Fig S1-S8+Table S1

## Data Availability

The datasets generated during the current study have been deposited into the National Center for Biotechnology Information under project numbers PRJNA598317 and PRJNA598307.
