# Supplementary material for: Melatonin-induced DNA demethylation of metal transporters and antioxidant genes alleviates lead stress in radish plants
Source: Hortic Res. 2021 Jun 1;8:124. doi: 10.1038/s41438-021-00561-8 (PMC8167184; doi:10.1038/s41438-021-00561-8)
Supplement: Supplementary file 1 — Supplementary data Fig S1-S8+Table S1 [file 41438_2021_561_MOESM1_ESM.pdf]

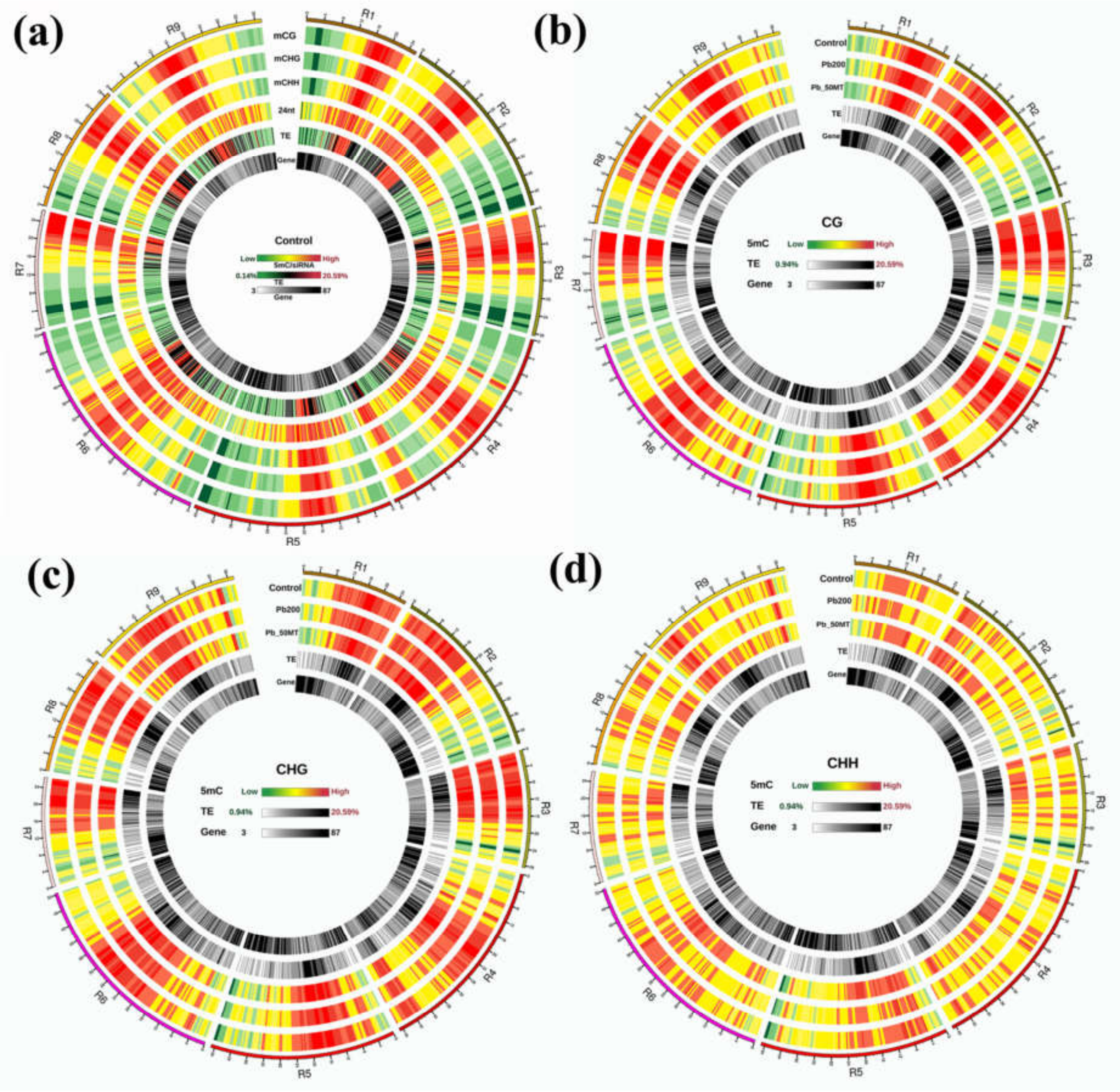

Fig. S1 Methylome features in radish. (A) The high-resolution map of DNA methylation in radish. (B-D) DNA methylome features of radish with or without Pb stress and MT treatment in CG (B), CHG (C) and CHH (D) contexts, respectively

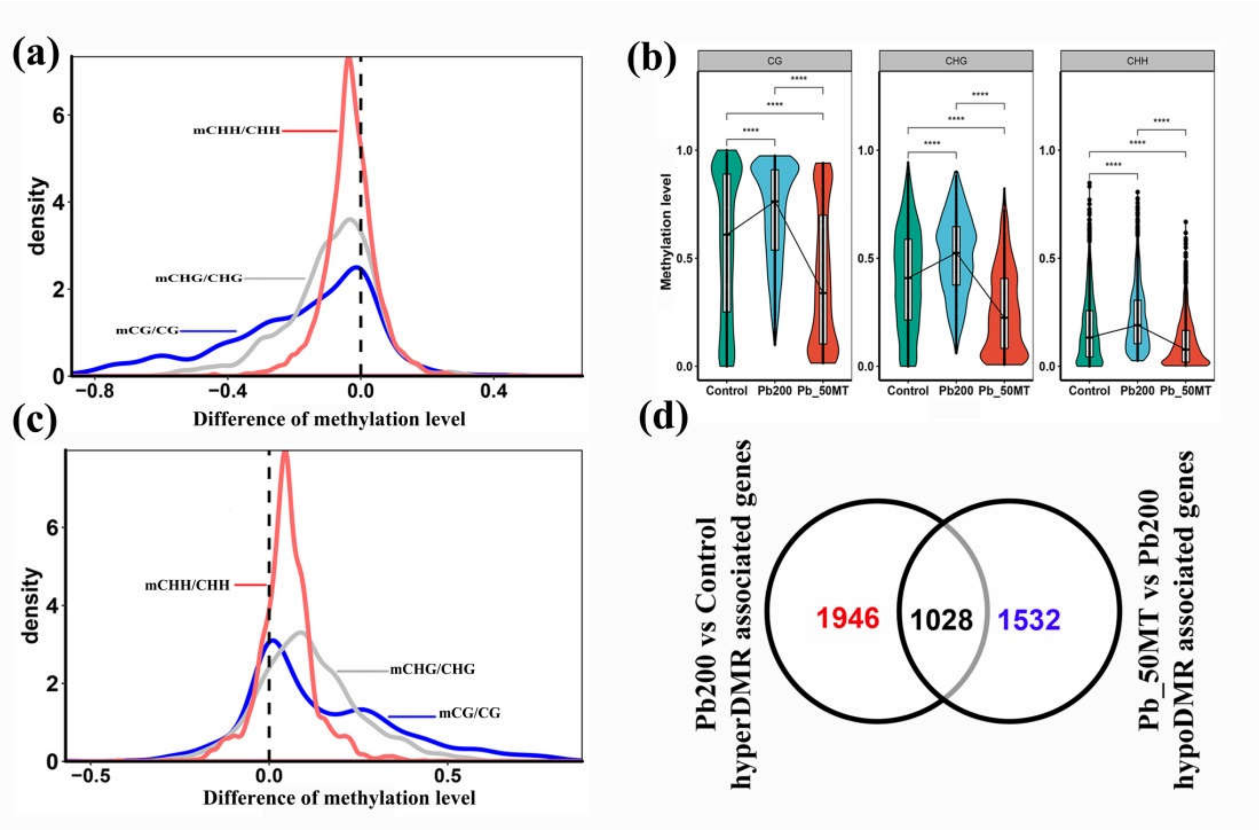

Fig. S2. **DNA methylation distribution of hyper-DMRs and hypo-DMRs.** (A) Kernel density plot of methylation change of Pb_50MT in Pb200 vs Control hyper-DMRs. (B) The DNA methylation distribution of Pb_50MT vs Pb200 hypo-DMRs in Control (C) Kernel density plot of methylation change of Control in Pb_50MT vs Pb200 hypo-DMRs. (D) The overlapped DMRs associated genes between Pb200 vs Control hyper-DMRs and Pb_50MT vs Pb200 hypo-DMR. *P-values* were calculated by study-tests, *, $P < 0.05$; **, $P < 0.01$; ***, $P < 0.001$; ns, not significant.

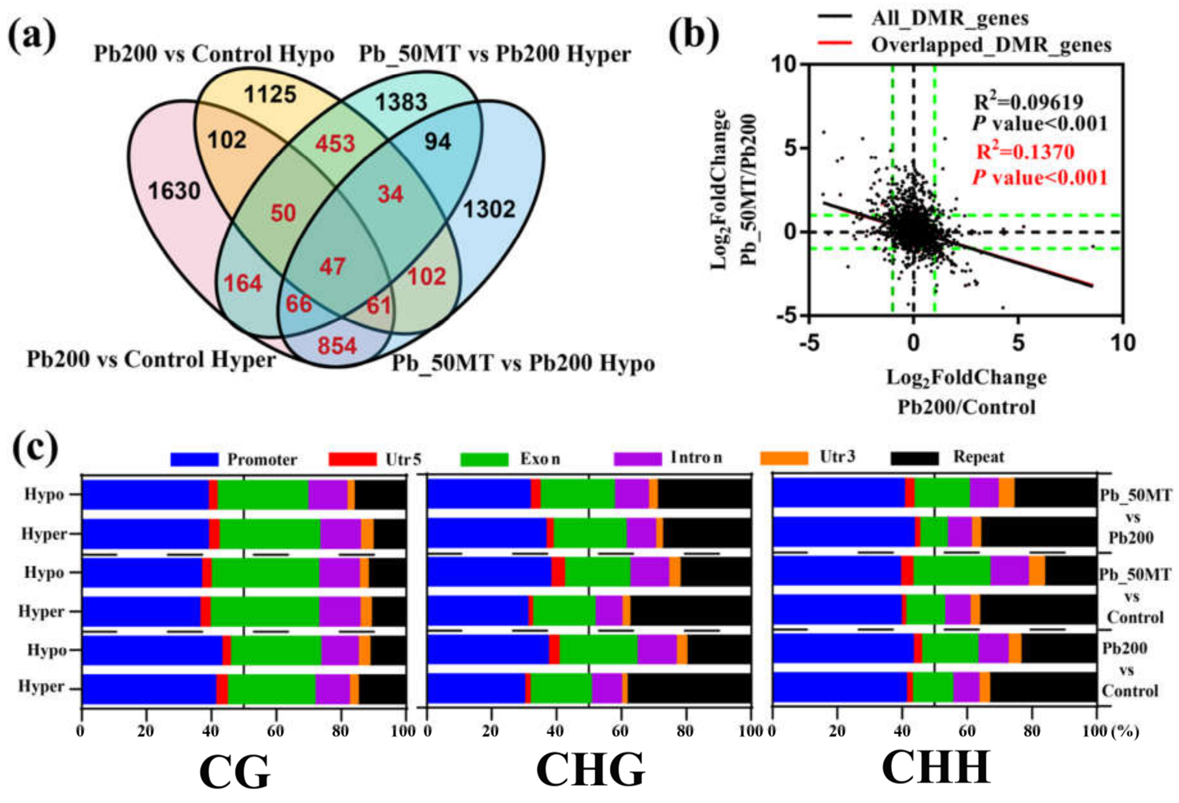

Fig. S3 The distribution of DMRs in different regions and correlation of DMR-associated genes between Pb200 vs Control and Pb_50MT vs Pb200. (a) Venn map of differentially methylated regions (DMRs) associated genes between Pb200 vs Control and Pb_50MT vs Pb200. **(b)** The relationship of differential expression level between Pb200 vs Control and Pb_50MT vs Pb200. **(c)** Relative proportions of different regions (Promoter, Utr5, Exon, Intron, Utr3 and Repeat) for the mCG, mCHG, and mCHH sequence contexts.

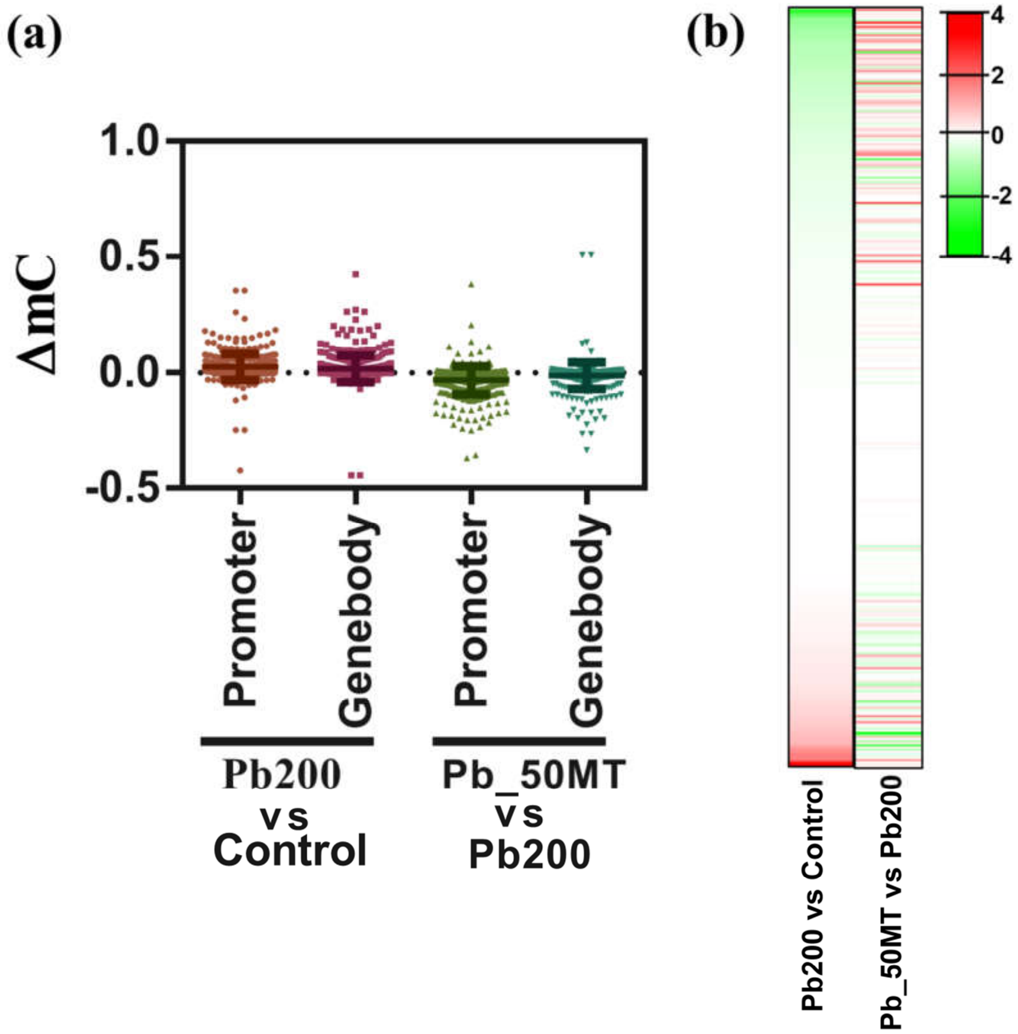

Fig. S4 The ion binding associated genes from GO terms of Pb_50MT vs Pb200 DMR-associated genes. **(a)** Differential methylation level of ion binding associated genes. **(b)** Expression level of ion binding associated genes.

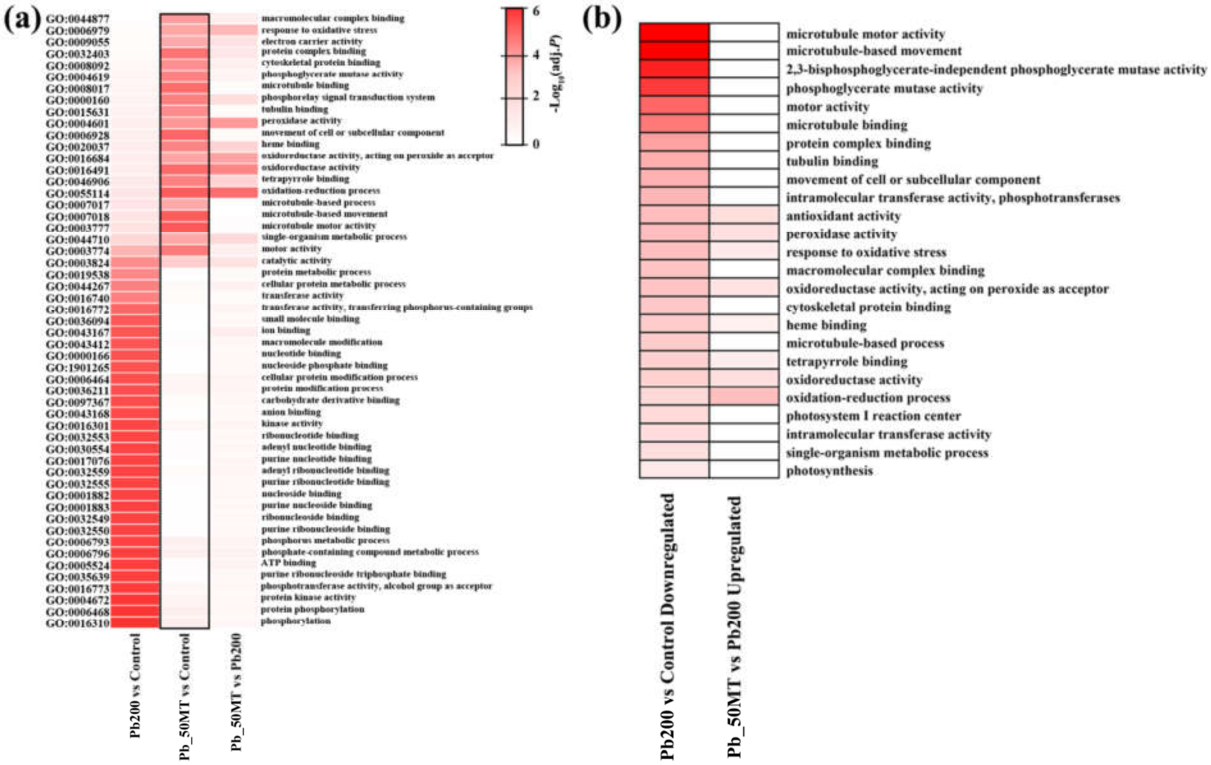

Fig. S5. GO terms of DEGs in Pb200_vs Control, Pb_50MT vs Control and Pb_50MT vs Pb200. (a) Significantly enriched GO terms of DEGs in Pb200_vs Control, Pb_50MT vs Control and Pb_50MT vs Pb200. **(b)** GO terms of downregulated DEGs in Pb200_vs Control and upregulated DEGs in Pb_50MT vs Pb200.

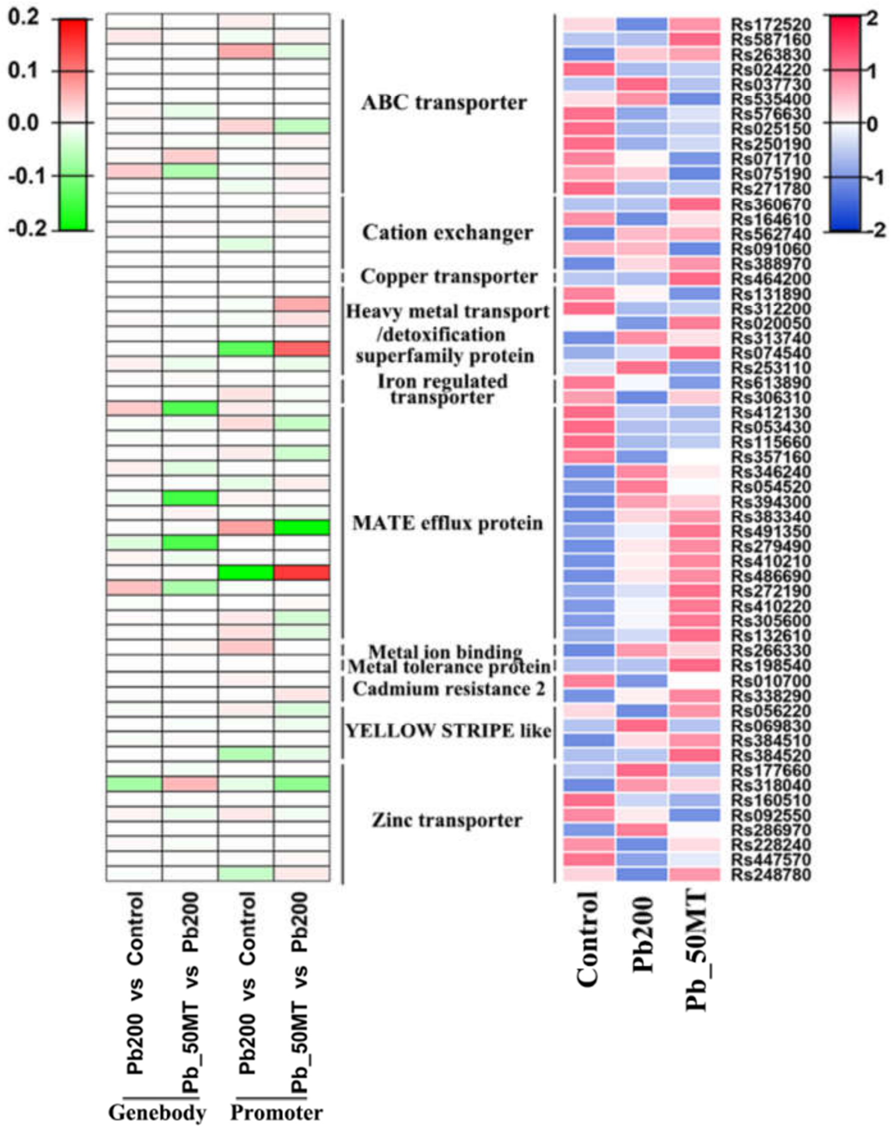

Fig. S6 The expression and methylation level of HM associated genes in response to Pb stress.

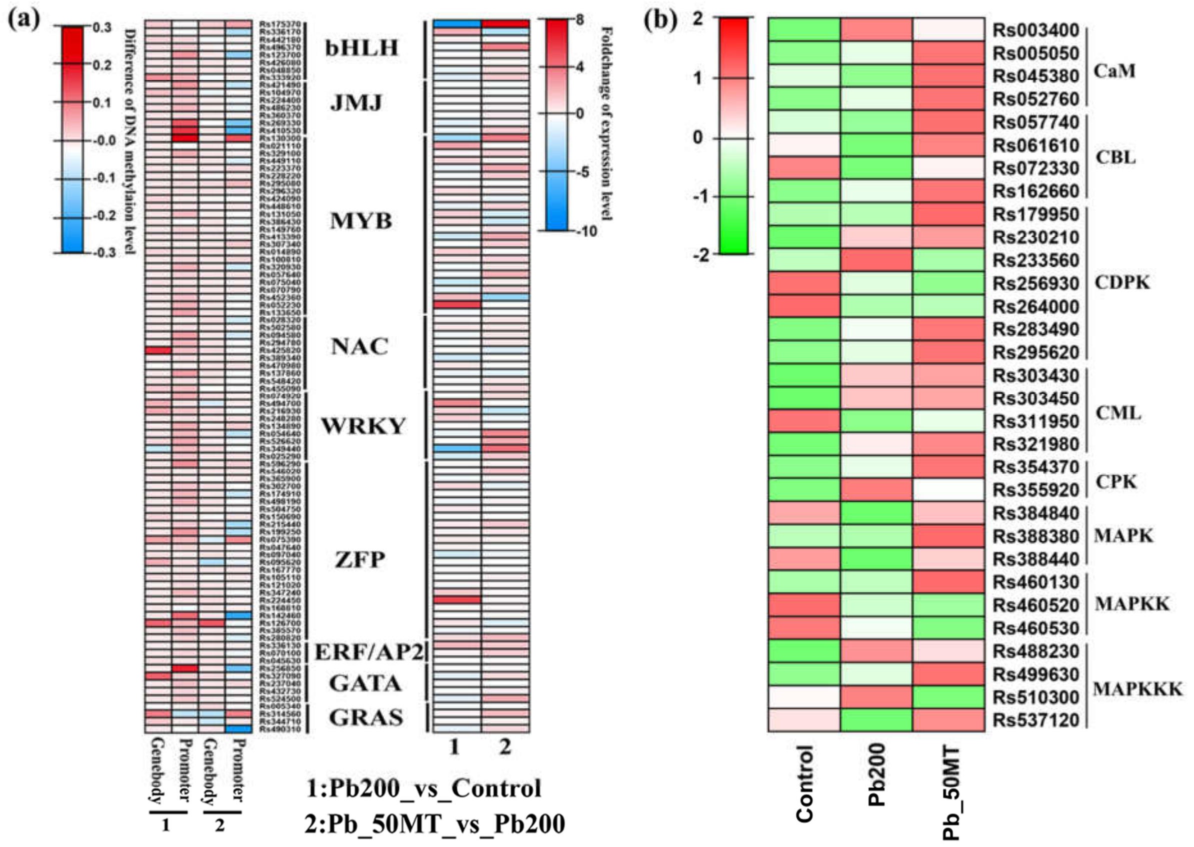

Fig. S7 Differential methylation and expression level of TFs and signaling transduction associated genes. (a) The differential methylation and expression level of DMR-associated TFs in Pb200_vs_Control and Pb_50MT_vs_Pb200. **(b)** The expression level of differentially expressed genes in calcium and MAPK signaling.

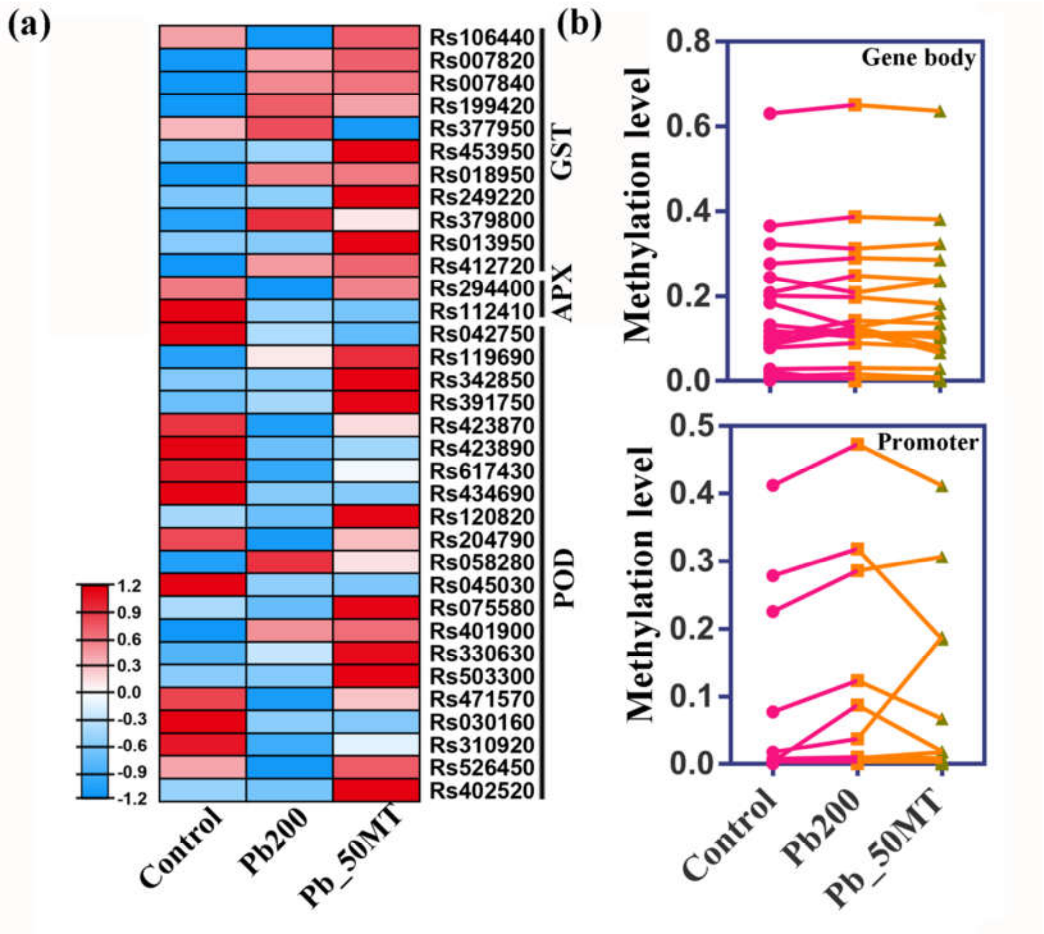

Fig. S8 The expression and methylation level of DEGs associated with antioxidant enzyme activities. (a) The expression level of DEGs associated with antioxidant enzyme activities in Control, Pb200 and Pb_50MT. **(b)** The methylation level of DEGs associated with antioxidant enzyme activities in promoter and gene body regions.

Table S1 The mapping statistics of bisulfite sequencing data.

Sample	C (Mb)	Raw_bases(G)	clean_bases(G)	Mapping rate(%)	Whole genome average coverage depth (X)	CG (Mb)	CHG (Mb)	CHH (Mb)	mC(Mb)	mC (%)	mCG(Mb)	mCG/CG (%)	mCG/mC(%)	mCHG(Mb)	mCHG/CHG (%)	mCHG/mC(%)	mCHH(Mb)	mCHH/CHH (%)	mCHH/mC(%)
Control1	1296.3	21.64	20.62	56.3	22.19	180.6	210.6	905	271.8	20.97	121.2	67.09	44.6	75.4	35.79	27.73	75.2	8.31	27.67
Control2	1186.8	20.65	19.5	56.5	21.33	182.5	192.1	812.2	291.2	24.54	134.8	73.84	46.3	71.2	37.08	24.46	85.2	10.49	29.25
Pb200_1	1137.3	20.11	19.04	55.97	20.42	171.1	184.8	781.3	291.3	25.61	127.8	74.70	43.9	78.8	42.62	27.04	84.6	10.83	29.05
Pb200_2	1019.6	17.54	16.62	56.23	18.36	153.3	165.9	700.3	239.6	23.5	110.9	72.37	46.3	60.6	36.51	25.28	68.1	9.72	28.41
Pb_50MT1	1338.8	22.76	21.66	55.68	23.07	196.1	220	922.7	328.3	24.52	137.3	69.99	41.8	86.5	39.30	26.34	104.5	11.33	31.85
Pb_50MT2	1357.2	23.18	21.97	54.71	22.7	195.3	224.7	937.3	281.1	20.71	130.7	66.90	46.5	73.6	32.74	26.17	76.9	8.20	27.34
